# The adenosine deaminase inhibitor erythro-9-[2-hydroxyl-3-nonyl]-adenine decreases intestinal permeability and protects against experimental sepsis: a prospective, randomised laboratory investigation

**DOI:** 10.1186/cc7033

**Published:** 2008-10-13

**Authors:** Nalan Kayhan, Benjamin Funke, Lars Oliver Conzelmann, Harald Winkler, Stefan Hofer, Jochen Steppan, Heinfried Schmidt, Hubert Bardenheuer, Christian-Friedrich Vahl, Markus A Weigand

**Affiliations:** 1 Department of Thoracic and Cardiovascular Surgery, University of Mainz, Langenbeckstr. 1, 55131 Mainz, Germany; 2 Department of Anesthesiology, University of Heidelberg, Im Neuenheimer Feld 110, 69120 Heidelberg, Germany; 3 Department of Anesthesiology and surgical Intensive Care Medicine, University hospital of Gießen and Marburg, Campus Gießen, Rudolf-Buchheim Strasse 7, 35292 Gießen, Germany

## Abstract

**Introduction:**

The treatment of septic conditions in critically ill patients is still one of medicine's major challenges. Cyclic nucleotides, adenosine and its receptors play a pivotal role in the regulation of inflammatory responses and in limiting inflammatory tissue destruction. The aim of this study was to verify the hypothesis that adenosine deaminase-1 and cyclic guanosine monophosphate-stimulated phosphodiesterase inhibition by erythro-9-[2-hydroxyl-3-nonyl]-adenine could be beneficial in experimental endotoxicosis/sepsis.

**Method:**

We used two established animal models for endotoxicosis and sepsis. Twenty-four male Wistar rats that had been given intravenous endotoxin (*Escherichia coli *lipopolysaccharide) were treated with either erythro-9-[2-hydroxyl-3-nonyl]-adenine infusion or 0.9% saline during a study length of 120 minutes. Sepsis in 84 female C57BL/6 mice was induced by caecal ligation and puncture. Animals were treated with repeated erythro-9-[2-hydroxyl-3-nonyl]-adenine injections after 0, 12 and 24 hours or 4, 12 and 24 hours for delayed treatment.

**Results:**

In endotoxaemic rats, intestinal production of hypoxanthine increased from 9.8 +/- 90.2 μmol/l at baseline to 411.4 +/- 124.6 μmol/l and uric acid formation increased from 1.5 +/- 2.3 mmol/l to 13.1 +/- 2.7 mmol/l after 120 minutes. In endotoxaemic animals treated with erythro-9-[2-hydroxyl-3-nonyl]-adenine, we found no elevation of adenosine metabolites. The lactulose/L-rhamnose ratio (14.3 versus 4.2 in control animals; p = 2.5 × 10^-7^) reflects a highly permeable small intestine and through the application of erythro-9-[2-hydroxyl-3-nonyl]-adenine, intestinal permeability could be re-established. The lipopolysaccharide animals had decreased L-rhamnose/3-O-methyl-D-glucose urine excretion ratios. Erythro-9-[2-hydroxyl-3-nonyl]-adenine reduced this effect. The mucosa damage score of the septic animals was higher compared with control and therapy animals (p < 0.05). Septic shock induction by caecal ligation and puncture resulted in a 160-hour survival rate of about 25%. In contrast, direct adenosine deaminase-1 inhibition resulted in a survival rate of about 75% (p = 0.0018). A protective effect was still present when erythro-9-[2-hydroxyl-3-nonyl]-adenine treatment was delayed for four hours (55%, p = 0.029).

**Conclusions:**

We present further evidence of the beneficial effects achieved by administering erythro-9-[2-hydroxyl-3-nonyl]-adenine, an adenosine deaminase-1 and cyclic guanosine monophosphate-stimulated phosphodiesterase inhibitor, in an endotoxicosis and sepsis animal model. This suggests a potential therapeutic option in the treatment of septic conditions.

## Introduction

Despite improvements in treatment modalities, the leading cause of death in non-coronary intensive care unit patients remains sepsis and septic shock, complex systemic activations of inflammation and coagulation in response to an infectious insult [1,2].

The purine nucleoside adenosine, a plurifunctional mediator and modulator of myriad physiological processes, which also serves as the substrate for ATP, is elevated at injured and inflamed sites, as well as in the plasma of septic and septic shock patients [3]. It is becoming increasingly apparent that this molecule and its receptors that elevate levels of cAMP, play a crucial role in the regulation of inflammatory responses and in limiting inflammatory tissue destruction [4-7]. By signalling through its specific G_s _protein-coupled A_2A _adenosine receptor, adenosine suppresses the immune system, primarily by inhibiting lymphoid or myeloid cells [5,8] including neutrophils [9], macrophages [10], lymphocytes [11,12] and platelets [13]. A_2A _receptor-knockout mice present a phenotype of enhanced tissue damage and inflammation [5,14]. Furthermore, adenosine is an endogenous inhibitor of neutrophil-induced endothelial cell injury [15,16] and β_2_-integrin expression on polymorphonuclear leucocytes, which mediate adhesion to the vascular endothelium, is mainly modulated by A_2A _receptors [17].

Inhibition of rephosphorylation of adenosine by adenosine kinase inhibitors [18] or its degradation by adenosine deaminase (ADA) improves survival of sepsis in various sepsis models [19-21]. ADA is an enzyme that is involved in purine metabolism and essential for the proliferation, maturation and function of lymphoid cells. Congenital deficiency of this enzyme is associated with an accumulation of deoxyadenosine triphosphates that will inhibit the activity of ribonucleotide diphosphate reductase. This results in severe combined immunodeficiency disease (SCID).

ADA activity is composed of two isoenzymes, referred to as ADA1 and ADA2 [22]. ADA1 is ubiquitous and highly efficient in deaminating the substrates adenosine and 2'deoxyadenosine. The isoenzyme ADA2 coexists with ADA1 only in monocytes and macrophages [23]. Law and colleagues demonstrated the beneficial effect of 2'-deoxycoformycin (pentostatin), an exclusive ADA2 inhibitor, in preventing the systemic inflammatory response syndrome secondary to faecal peritonitis in rats [24]. There is a lack of data concerning the question if a specific inhibitor of ADA1 could also influence survival rates in septic conditions.

Another critical aspect of a septic condition is its intestinal barrier dysfunction resulting in bacterial translocation and thereby perpetuating and aggravating the syndrome [25,26]. Endothelial hyperpermeability which results in a vascular leakage can induce edema formation in the intestinal mucosa. This might contribute to increased gut permeability. Suttorp and Seybold identified the importance of cyclic guanosine monophosphate-stimulated phosphodiesterase-2 (PDE2) for the integrity of endothelial barrier function [27,28]. They presented evidence that in severe infection, high PDE2 activity may contribute to endothelial barrier dysfunction, which can be antagonised by PDE2 inhibition [28].

In this study we used an endotoxicosis animal model and a sepsis animal model to provide evidence of the beneficial effects of administration of erythro-9-[2-hydroxy-3-nonyl] adenine (EHNA), a specific ADA1 and PDE2 inhibitor, on the production of adenosine metabolites and intestinal permeability, and improved survival rates.

## Materials and methods

All experiments were performed in accordance with the guidelines for research with experimental animals (Helsinki Declaration) and were approved by the Governmental Animal Protection Committee (Karlsruhe, Germany).

### Endotoxaemic challenge

Male Wistar rats (250 g to 330 g body weight) were kept on a diet of standard rat food until the day before the experiment. Eight hours before the experiment began, food was withheld from all animals but free access to water was maintained. The rats were anaesthetised intraperitoneally with 60 mg/kg sodium pentobarbital (Nembutal, Sanofi-aventis, Duesseldorf, Germany). The right internal jugular vein, the left femoral vein and the left femoral artery were cannulated with polyethylene tubings (outer diameter = 0.9 mm; inner diameter = 0.5 mm) to measure mean arterial pressure, and to allow drug infusion and blood sampling, respectively. For blood sampling from the portal vein, a midline laparotomy was performed, the small intestine was carefully displaced and the portal vein was punctured proximal to the splenic vein at three different times. After each blood collection from the portal vein, the intestine was covered with warmed (37°C) saline-soaked gauze to preserve moistness and temperature. Rectal temperature was measured using a thermistor probe (YSI-400 Series) and maintained at 37°C with the help of a heating ventilator.

**Figure 1:**
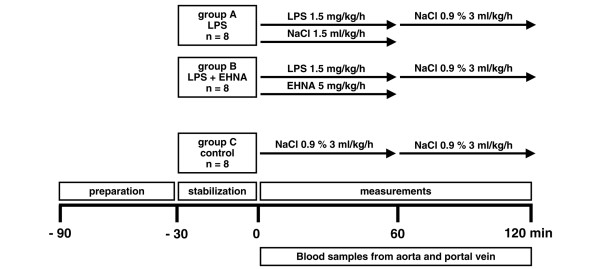
**Endotoxaemic challenge (experimental design)**. Rats were randomised to three groups of eight animals each. After preparation, a 30 minute stabilisation period was allowed. The animals of group B (lipopolysaccharide (LPS) + erythro-9-[2-hydroxyl-3-nonyl]-adenine (EHNA)) received 5 mg/kg/hour EHNA intravenously as a continuous infusion over one hour. Endotoxaemia was induced immediately after baseline measurements by continuous intravenous infusion of LPS for 60 minutes. Animals of the control group received no EHNA or LPS. The same amount of fluids was infused in all rats for the total duration of the experiment (120 minutes).

Rats were randomised into three groups of eight animals each (Figure 1). After the animals were prepared, they were allowed a 30-minute stabilisation period. Endotoxaemia was induced immediately after the baseline measurements by continuous intravenous infusion of 1.5 mg/kg/hour endotoxin (lipopolysaccharide (LPS) from *Escherichia coli *026:B6; Sigma Chemicals, Deisenhofen, Germany) diluted in sodium chloride (NaCl) 0.9% for 60 minutes. The animals of group B (LPS + EHNA) additionally received a continuous intravenous infusion of 5 mg/kg/hour EHNA diluted in NaCl 0.9% for 60 minutes from the beginning of the endotoxaemic challenge. Animals of the control group received no EHNA or endotoxin. The same amount of fluids was infused in all rats for the total duration of the study (120 minute).

### Analysis of purine compounds

Purine compounds (hypoxanthine and uric acid) were measured in 0.2 ml of collected blood in precooled dipyridamole solution (0.2 ml; 5 × 10^-5 ^M) to prevent nucleoside uptake by red blood cells. After immediate centrifugation at 4°C, plasma supernatant (0.3 ml) was deproteinated with perchloric acid (70%; 0.05 ml). After neutralisation with potassium dihydrogen phosphate (KH_2_PO_4_) and centrifugation, nucleosides were determined by HPLC. We automatically injected 0.1 ml samples onto a C-18 column (Nova-Pak C18, 3.9 mm × 150 mm, Waters Instruments, Rochester, NY). The linear gradient started at 100% KH_2_PO_4_/K_2_HPO_4 _(1:1 mixture of mono and dipotassium phosphate) 1:1 (0.1 M; pH 4.0) and increased to 60% of 60/40 methanol/water (v/v) in 15 minutes, the flow rate being 1.0 ml/minute. This was followed by a reversal of the gradient to initial conditions over the next three minutes. We continuously monitored absorbance of the column eluate by using a photodiode array detector (Waters 996) to measure hypoxanthine at 254 nm and uric acid at 293 nm. We performed peak identification and quantitation of the respective compounds by comparing the retention times of the sample peaks with respective peaks of ultrapure standards.

### Measurements

Mean arterial blood pressure and temperature were recorded at baseline and at 15, 30, 45, 60, 75, 90, 105 and 120 minutes after starting the endotoxin or saline infusion. Hypoxanthine and uric acid were determined from arterial and portal venous blood samples taken at baseline, 60 and 120 minutes later. We based our calculation of the quantity of purine compounds produced by the intestine on the difference between portal venous and arterial concentrations.

### Assessment of intestinal permeability and absorption

Timed recovery of 3-O-methyl-D-glucose, lactulose and L-rhamnose in urine after duodenal administration was assessed in our endotoxaemic rats in order to estimate absorptive capacity and intestinal permeability. In brief, after the animals were prepared as described above and the LPS infusion was started, the rats received 3 ml of a solution containing 25 g/l 3-O-methyl-D-glucose (Sigma-Aldrich Chemie GmbH, Munich, Germany), 25 g/l lactulose (Sigma-Aldrich Chemie GmbH, Munich, Germany) and 10 g/l L-rhamnose (Sigma-Aldrich Chemie GmbH, Munich, Germany) direct into the duodenum after puncturing the proximal part of the organ. Urine was collected after animals were euthased by puncturing the urinary bladder. High performance HPLC was conducted according to the procedure described by Sorensen and colleagues [29]. Preabsorption factors, such as dilution by secretion and intestinal transit time, and postabsorption factors, such as systemic distribution and renal clearance, are assumed to affect the saccharides equally. Therefore, the urinary excretion rhamnose/glucose and lactulose/rhamnose ratios are considered as parameters for intestinal absorption capacity and permeability, respectively [29,30].

### Evaluation of intestinal mucosal damage

After the animals were sacrificed, segments of the distal ileum 3 to 5 cm in length were cautiously exteriorised and immediately snap frozen in liquid nitrogen. The frozen ileal mucosa samples were cut into 4 μm thick sections using a cryostat (Leica CM1850, Leica Microsystems, Wetzlar, Germany), then mounted on super frost slides, air dried at 37°C, overnight and stained with haematoxylin and eosin following standard procedures. Mucosal damage grading was assessed by two independent observers according to the procedures described by Chiu and colleagues [31] (Tab. 1).

**Table 1:** Intestinal mucosal damage grading score.

Grade	Histological characteristics
Grade 0	Normal mucosal villi

Grade 1	Subepithelial Gruenhagen's space (oedema), usually at the apex of the villus

Grade 2	Extension of the subepithelial space with moderate lifting of epithelial layer from the lamina propria

Grade 3	Massive epithelial lifting down the sides of villi. A few tips may be denuded

Grade 4	Denuded villi with lamina propria and dilated capillaries exposed

Grade 5	Digestion and disintegration of lamina propria; haemorrhage and ulceration

### Caecal ligation and puncture

Caecal ligation and puncture (CLP) was performed as described previously [32-36]. In brief, female C57BL/6 mice aged 12 to 16 weeks were anaesthetised by intraperitoneal administration of 75 mg/kg Ketamine (Ketanest, Pfizer Pharma, Karlsruhe, Germany) and 16 mg/kg Xylazine (Rompun, Bayer AG, Leverkusen, Germany) in 0.2 ml sterile pyrogen-free saline (Braun AG, Melsungen, Germany). The caecum was exposed through a 1.0 to 1.5 cm abdominal midline incision and subjected to a ligation 6 mm from the caecal tip followed by a single puncture with a G23 needle. A small amount of stool was expelled from the punctures to ensure patency. The caecum was returned into the peritoneal cavity and the abdominal incision was closed by layers with 5/0 prolene thread (Ethicon, Norderstedt, Germany). No antibiotics were administered in this model. For the sham-operated mice serving as controls, the caecum was mobilised but no ligation or puncture was performed.

In order to investigate the therapeutic effect of EHNA, 10 mg/kg of the adenosine deaminase inhibitor was administered by intraperitoneal injection after 0, 12 and 24 hours or 4, 12 and 24 hours. Control groups received the same volume of LPS-free 0.9% NaCl solution. CLP was performed blind with respect to the identity of the treatment group. Survival after CLP was assessed four to six times a day for seven days.

### Statistical analysis

Data were analysed using the R language and environment for statistical computing and graphics (version 2.7.2) [37]. Data are presented in one dimensional dot plots, as well as mean and standard error of the mean (SEM) or using Kaplan-Meier survival curves. We performed Bartlett's test for homogeneity of variances. The differences between groups were assessed by one-way analysis of variance (ANOVA), post hoc Tukey-Kramer method for pairwise comparisons and log-rank-test for survival curve analysis. p < 0.05 were considered significant.

## Results

### Purine compounds

At the beginning of the experiment, mean arterial pressure and temperature showed no differences between groups and remained stable throughout the observation period in all groups (Table 2). The haemodynamic parameters of experimental animals are shown in Table 3.

**Table 2:** Mean arterial pressure and rectal temperature of endotoxaemic rats: Mean arterial pressure (MAP) and rectal temperature (temp) in control animals, in animals receiving 1.5 mg/kg endotoxin over a 60 minute period (lipopolysaccharide (LPS) group), and in animals receiving endotoxin plus an infusion of 5 mg/kg/hour erythro-9-[2-hydroxyl-3-nonyl]-adenine (EHNA) (LPS + EHNA group). Data are mean ± standard error of the mean.

Parameter	Group	Time (minutes)								
		0	15	30	45	60	75	90	105	120

MAP	Control	76.0 ± 1.6	76.5 ± 3.5	76.9 ± 3.7	82.1 ± 3.6	83.0 ± 3.6	79.6 ± 3.8	84.8 ± 2.9	83.4 ± 4.0	81.3 ± 4.1

	LPS	78.8 ± 3.0	73.6 ± 3.9	82.4 ± 4.4	83.1 ± 4.9	90.4 ± 3.8	87.0 ± 6.5	86.8 ± 4.7	84.8 ± 4.3	81.6 ± 4.8

	LPS + EHNA	77.4 ± 3.4	75.5 ± 2.7	76.6 ± 2.5	82.0 ± 2.8	83.5 ± 3.1	86.8 ± 2.9	90.3 ± 3.1	85.5 ± 3.3	83.6 ± 3.4

Temp	Control	36.1 ± 0.3	35.8 ± 0.5	36.3 ± 0.4	36.4 ± 0.5	36.4 ± 0.3	36.3 ± 0.3	36.8 ± 0.3	36.9 ± 0.3	36.6 ± 0.3

	LPS	36.8 ± 0.4	36.8 ± 0.5	37.0 ± 0.6	36.8 ± 0.4	37.3 ± 0.6	36.7 ± 0.6	36.7 ± 0.6	36.8 ± 0.5	36.8 ± 0.4

	LPS + EHNA	36.4 ± 0.3	36.9 ± 0.3	37.8 ± 0.2	38.2 ± 0.2	37.9 ± 0.1	37.0 ± 0.3	37.7 ± 0.3	37.8 ± 0.2	37.4 ± 0.2

**Table 3:** Haemodynamic parameters of endotoxaemic rats. art = arterial; BE = base excess; ENHA = erythro-9-[2-hydroxyl-3-nonyl]-adenine; LPS = lipopolysaccharide; pCO_2 _= partial pressure of carbon dioxide; pHCO_3_^- ^= bicarbonate; pO_2 _= partial pressure of oxygen; SO_2 _= oxygen saturation. Data are mean ± standard error of the mean.

Parameter	Group	Time (minutes)		
		0	60	120

art. SO_2_	Control	94.5 ± 0.7	97.2 ± 0.5	95.5 ± 1.0

	LPS	96.3 ± 0.8	97.4 ± 0.5	97.2 ± 0.3

	LPS + EHNA	94.4 ± 0.5	92.4 ± 4.3	91.3 ± 5.6

art. PO_2_	control	83.4 ± 4.4	86.7 ± 3.7	88.3 ± 2.8

	LPS	95.1 ± 9.5	107.1 ± 9.4	99.7 ± 4.2

	LPS + EHNA	75.7 ± 2.3	89.5 ± 5.3	86.6 ± 6.9

art. PCO_2_	control	47.2 ± 1.4	41.3 ± 1.7	40.2 ± 1.1

	LPS	42.8 ± 1.6	37.1 ± 1	35.0 ± 0.8

	LPS + EHNA	49.5 ± 1	35.8 ± 3.9	34.0 ± 2.4

art. HCO_3 _^-^	control	26.9 ± 0.5	24.5 ± 1.0	24.1 ± 0.5

	LPS	25.3 ± 1.0	22.8 ± 0.6	18.6 ± 1.0

	LPS + EHNA	28.2 ± 0.5	21.3 ± 3.4	18.7 2.5

art. PH	control	7.37 ± 0.01	7.40 ± 0.01	7.39 ± 0.01

	LPS	7.38 ± 0.01	7.40 ± 0.01	7.35 ± 0.02

	LPS + EHNA	7.36 ± 0.01	7.28 ± 0.12	7.31 ± 0.07

art. BE	control	1.4 ± 0.6	0.2 ± 1.0	0.2 ± 0.4

	LPS	0.5 ± 0.9	-1.1 ± 0.7	-5.8 ± 1.3

	LPS + EHNA	2.4 ± 0.6	-5.3 ± 5.9	-6.6 ± 3.7

**Figure 2:**
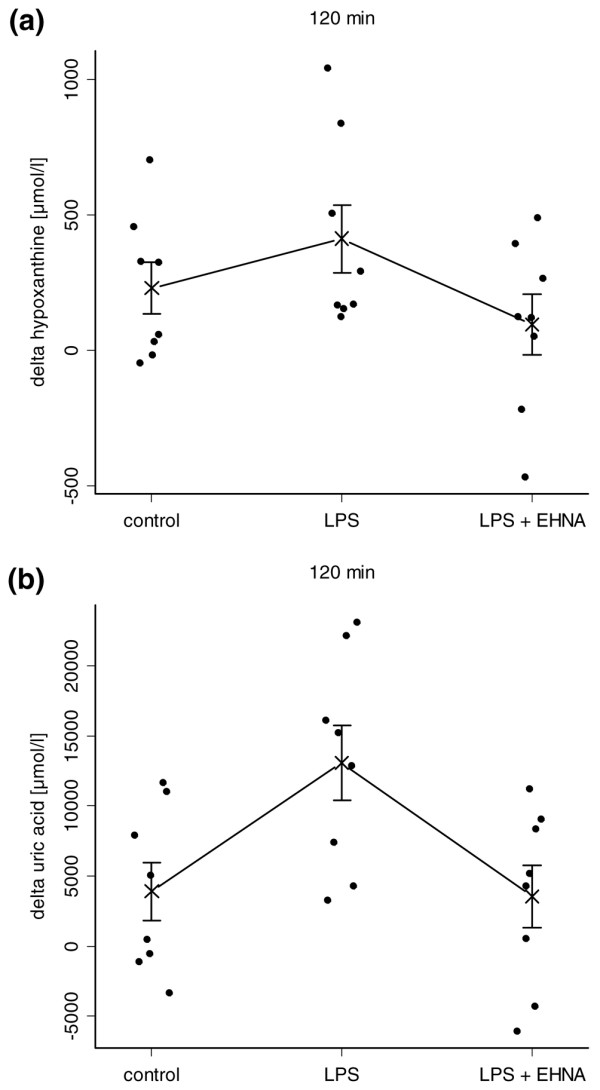
**Adenosine deaminase-1 inhibition prevents lipopolysaccharide (LPS)-induced intestinal hypoxanthine and uric acid formation**. (a) Intestinal release of hypoxanthine and (b) uric acid calculated as the differences (Δ) between portal venous and arterial concentrations of the purine metabolites after 120 minutes; in control animals, in animals receiving 1.5 mg/kg endotoxin over a 60 minute period (LPS group) and in animals receiving endotoxin plus an infusion of 5 mg/kg/hour erythro-9-[2-hydroxyl-3-nonyl]-adenine (EHNA) at the beginning of the endotoxin challenge (LPS + EHNA group). Data presented in one dimensional dot plots as well as mean and standard error of the mean (SEM). After 120 minutes the LPS group differed in the mean of intestinal hypoxanthine and uric acid production from control and EHNA-treated animals. (a) hypoxanthine production, analysis of variance (ANOVA) p = 0.02, post hoc Tukey-Kramer test p = 0.03; (b) uric acid production, ANOVA p = 0.01, post hoc Tukey-Kramer test p = 0.009.

Bartlett's test for all experimental groups revealed homogeneity of variances. In the control animals, the intestinal hypoxanthine and uric acid production remained statistically unchanged throughout the observation period (one-way ANOVA: hypoxanthine p = 0.6, uric acid p = 0.6). Similarly, the intestinal hypoxanthine and uric acid production in endotoxin-stimulated animals with EHNA application (LPS + EHNA group) did not change during the duration of the experiment (one-way ANOVA: hypoxanthine p = 0.07, uric acid p = 0.9). In contrast, in the endotoxaemic rats without EHNA application (LPS group), the intestinal production of hypoxanthine increased from 9.8 ± 90.2 μmol/l at baseline to 411.4 ± 124.6 μmol/l after 120 minutes (post hoc Tukey-Kramer test: p = 0.03), and the intestinal production of uric acid increased from 1.5 ± 2.3 mmol/l at baseline to 13.1 ± 2.7 mmol/l after 120 minute (post hoc Tukey-Kramer test: p = 0.01). Furthermore, after 120 minutes the LPS group differed in the mean of intestinal hypoxanthine and uric acid production from control and EHNA treated animals (hypoxanthine production: ANOVA p = 0.02, post hoc Tukey-Kramer test p = 0.03; uric acid production: ANOVA p = 0.01, post hoc Tukey-Kramer test p = 0.009) (Figures 2a and 2b).

### Intestinal permeability and absorption capacity

**Figure 3:**
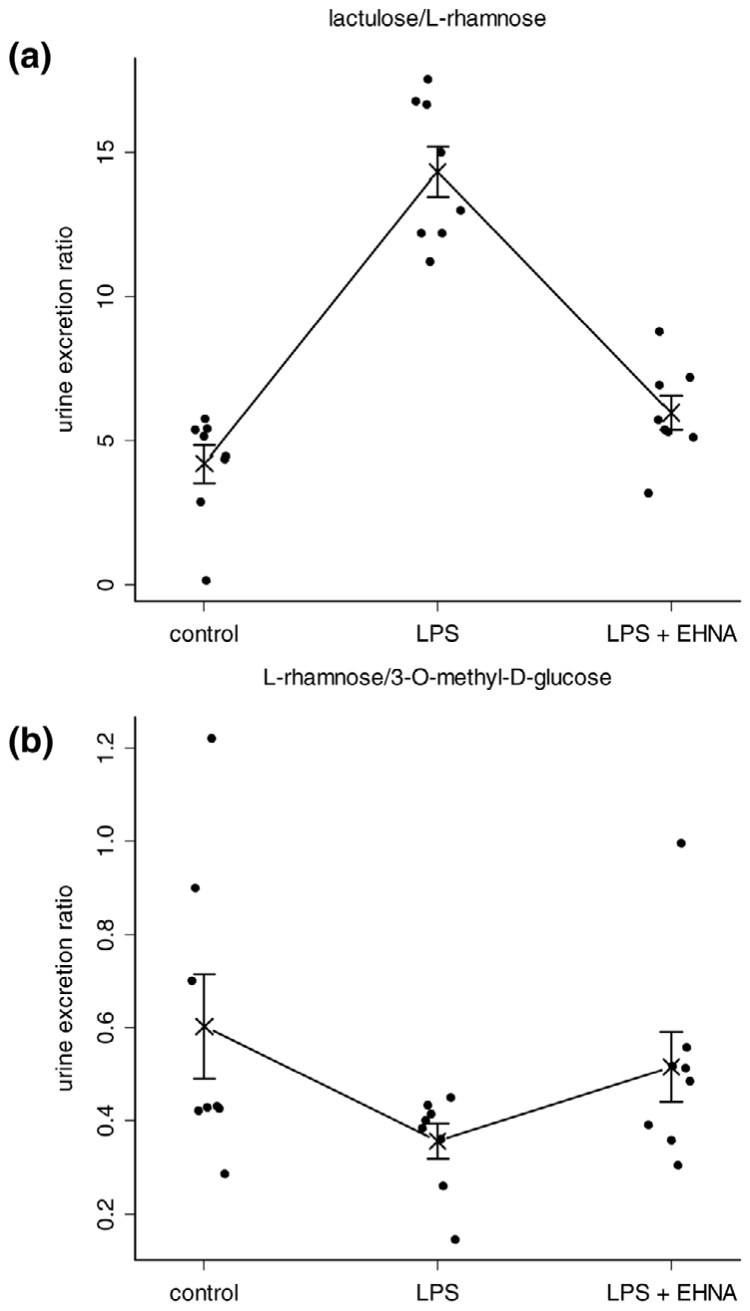
**Erythro-9-[2-hydroxyl-3-nonyl]-adenine (EHNA) administration re-establishes intestinal barrier as well as absorption capacity: Recovery of 3-O-methyl-D-glucose, lactulose and L-rhamnose in urine after direct duodenal administration was measured**. (a) The lactulose/L-rhamnose ratio of the lipopolysaccharide (LPS) group was about three times higher than the control group, which indicates a highly permeable small intestine in septic rats. Through the application of EHNA the intestinal permeability could be re-established to a value comparable with control animals (analysis of variance (ANOVA) p = 3.5 × 10^-9^, Tukey-Kramer test p = 2 × 10^-7^). (b) LPS animals had decreased L-rhamnose/3-O-methyl-D-glucose urine excretion ratios (0.38 ± 0.05) compared with normal controls (0.58 ± 0.12, post hoc test p = 0.05), consistent with a decrease in the gastrointestinal functional absorptive capacity. ADA1 inhibition with a single dose of EHNA diminished this effect. Data presented in one dimensional dot plots as well as mean and standard error of the mean (SEM).

The recovery of saccharides excreted in urine at their appropriate ratios is shown in Figure 3. The lactulose/L-rhamnose ratio of the LPS group with an elevation of about three times the value of the control group (ANOVA p = 3.5 × 10^-9^, Tukey-Kramer test p = 2 × 10^-7^) reflects a highly permeable small intestine in septic rats. Through the application of EHNA, intestinal permeability could be recovered to a value comparable with that of control animals (Figure 3a). Also, the LPS animals had decreased L-rhamnose/3-O-methyl-D-glucose urine excretion ratios (0.38 ± 0.05) compared with normal controls (0.58 ± 0.12, post hoc test p = 0.05), consistent with a decrease in gastrointestinal functional absorptive capacity. ADA1 inhibition by a single dose of EHNA diminished this effect (Figure 3b).

### Evaluation of intestinal mucosal damage

**Figure 4:**
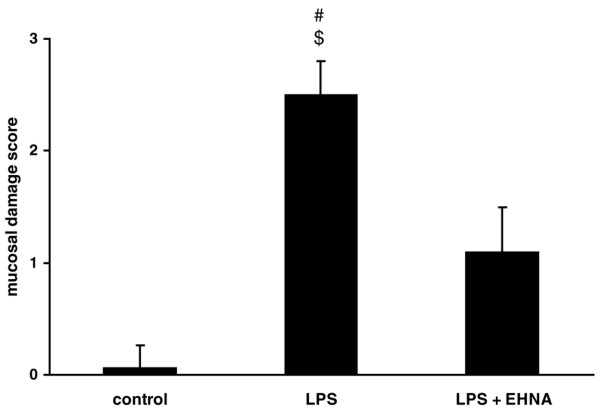
**Adenosine deaminase inhibition protects against intestinal mucosal damage during endotoxaemia**. Mucosal damage grading was assessed [31]. Data are mean ± standard error of the mean (SEM). # p < 0.05 versus control; $ p < 0.05 versus lipopolysaccharide (LPS) + erythro-9-[2-hydroxyl-3-nonyl]-adenine (EHNA).

**Figure 5:**
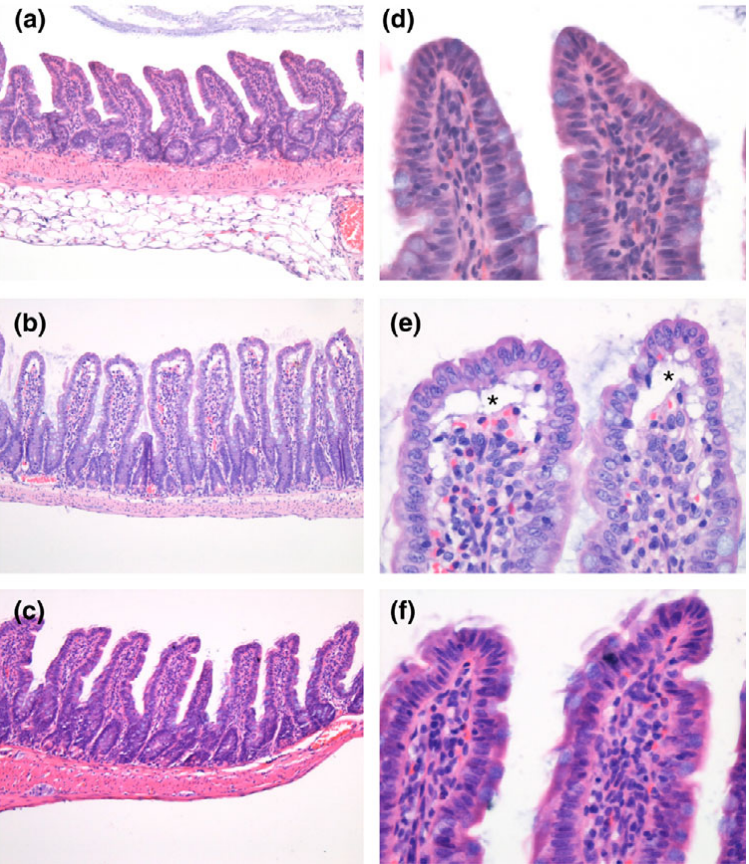
**Adenosine deaminase inhibition protects against intestinal mucosal damage during endotoxaemia**. Representative microphotographs of haematoxylin & eosin (H & E) stained sections of the terminal ileum of experimental groups. (a,d) Control group with normal appearance of small intestinal mucosa with long villi that have occasional goblet cells, small and basal located nuclei of epithelial cells, and a normal lamina propria. (b, e) Lipopolysaccharide (LPS) group with disturb mucosal architecture showing plump villi with markedly increased villous stroma, a lifting of epithelial layer from the lamina propria (*subepithelial Gruenhagen's space), and a higher nucleus-plasma ratio of epithelial cells. (c, f) LPS + erythro-9-[2-hydroxyl-3-nonyl]-adenine (EHNA) group with a similar appearance of small intestinal mucosa as in the control group. (a-c) original magnification of ×16 and (d-F) ×64

Histologically, we were able to demonstrate a protective effect of ADA1 inhibition by EHNA against intestinal mucosal damage in our endotoxaemic animal model (Figures 4 and 5). According to an established mucosal damage score [31], the control and therapy groups (LPS + EHNA) are not statistically different even though the score is somewhat increased in the therapy group. In contrast, the mucosa damage score of the septic animals is higher compared with control and therapy animals (p < 0.05).

### Survival after CLP

**Figure 6:**
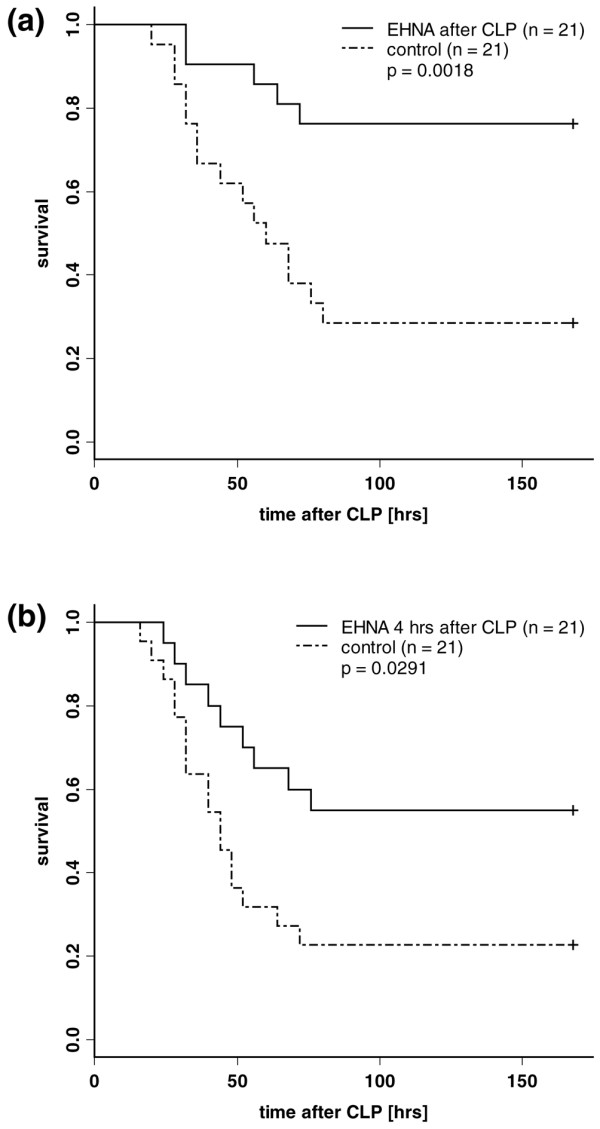
**Adenosine deaminase 1 inhibition protects against septic shock induced by caecal ligation and puncture (CLP)**. Kaplan-Meier survival curves; septic shock induction by CLP resulted in a 160-hour survival rate of about 25%. In contrast, direct adenosine deaminase-1 inhibition after septic shock induction via CLP resulted in a 160-hour survival rate of about 75% (p = 0.0018). A protective effect was still present when the erythro-9-[2-hydroxyl-3-nonyl]-adenine (EHNA) treatment was delayed for four hours after CLP (55% survival, p = 0.029).

Septic shock induction by CLP resulted in a 160-hour survival rate of about 25%. In comparison, the direct adenosine deaminase-1 inhibition after septic shock induction via CLP resulted in a 160-hour survival rate of about 75% (p = 0.0018). A protective effect was still present when the treatment of EHNA was delayed for four hours after CLP (55% survival, p = 0.029). Kaplan-Meier survival curves are shown in Figure 6.

## Discussion

Adenosine and its receptors play a crucial role in the regulation of inflammatory responses and in limiting inflammatory tissue destruction [4-6]. Elevation of adenosine and activation of its receptors and their downstream signalling are promising targets for treatment of septic conditions [38]. Thiel and colleagues showed that intravenous infusion of adenosine during endotoxaemia protects from oxygen-mediated tissue injury without compromising the bactericidal mechanisms of polymorphonuclear leucocytes [39]. In further studies, the authors demonstrate that the A_2A _receptor agonist compensated for the loss of endogenously formed adenosine in inflamed lungs of oxygenated mice and thereby prevented inflammatory lung injury and death [40]. The inhibition of the degradation of adenosine by ADA improves survival from sepsis [19-21]. ADA activity is composed of the two isoenzymes ADA1 and ADA2 [22]. ADA2 coexists with ADA1 only in monocytes and macrophages [23]. The specific ADA2 inhibitor 2'-deoxycoformycin (pentostatin), primarily used to treat hairy cell leukaemia, has seen increasing attention as an immunosuppressant [41]. Law and colleagues demonstrated the beneficial effect of pentostatin application in preventing the systemic inflammatory response syndrome secondary to faecal peritonitis in rats [24]. On the other hand, ADA1 is ubiquitous and highly efficient in deaminating the substrates adenosine and 2'deoxyadenosine.

In addition, a hallmark of septic conditions are their intestinal barrier dysfunctions resulting in bacterial translocation and thereby perpetuating and aggravating the syndrome [25,26]. Endothelial cells are important mediators in orchestrating the host response in sepsis [42]. A pivotal feature of sepsis is microvascular dysfunction in which endothelial activation, dysfunction and thereby hyperpermeability seem to play a central role [43]. Endothelial hyperpermeability results in a vascular leakage of the intestinal mucosa that might contribute to increased gut permeability. Suttorp and Seybold identified the importance of cyclic guanosine monophosphate-stimulated PDE2 for the integrity of the endothelial barrier function [27,28]. They presented evidence that in severe infection, high PDE2 activity may contribute to endothelial barrier dysfunction, which can be antagonised by PDE2 inhibition [28].

We based our approach on the hypothesis that ADA1 and PDE2 inhibition, targeting monocytes and the endothelium/intestinal epithelium respectively, could be beneficial in experimental septic conditions and employed EHNA, a specific ADA1 and PDE2 inhibitor, as the therapeutic agent.

There are numerous animal models and all of them have limitations and advantages. Indeed there is controversy whether endotoxaemic shock and sepsis are different entities or not. However, the LPS model has a role in helping to understand the sepsis phenotype [44]. As our experimental basis, we utilised this commonly used endotoxicosis model. LPS-induced endotoxaemic shock simplifies aspects of experimental design while maintaining features of a compensated human sepsis (such as hypermetabolism, anorexia, mild hypotension, leucocytosis and hyperlactataemia [45,46]). Furthermore doses of LPS are readily measured and controlled because it is a stable and relatively pure compound. This ensures reproducibility of the septic challenge. As shown by Schmidt and colleagues, this endotoxaemic rat model is associated with a release of purine metabolites from the intestinal tract during endotoxaemia [47]. In our endotoxaemic rats, the intestinal production of hypoxanthine and uric acid was also increased. In contrast, in endotoxaemic animals treated with the ADA1/PDE2 inhibitor EHNA, an increased intestinal production was not observed, neither for hypoxanthine or uric acid. Increased serum uric acid correlates with severe sepsis and septic shock [48]. In addition, serum uric acid levels correlated significantly with scores from Acute Physiology and Chronic Health Evaluation (APACHE) II in critically ill patients [49,50]. Uric acid is a principal endogenous danger signal and is released from injured cells. Shi and colleagues demonstrated that by eliminating uric acid the immune response to antigens associated with injured cells is inhibited [51].

The data of Johnston and van Nieuwenhoven demonstrated that patients with acute sepsis exhibit an increased intestinal permeability (lactulose/rhamnose urinary excretion ratio) and a decreased intestinal absorption capacity (rhamnose/glucose urinary excretion ratio) compared with healthy control subjects [52,53]. In our study, the values for intestinal permeability and absorption capacity as a measure of an epithelial dysfunction of endotoxaemic animals treated with EHNA were comparable with the control rats. In contrast, the endotoxaemic animals presented a disturbed intestinal permeability and absorption capacity. Our assumption that the stabilisation of the intestinal barrier might be the result of endothelial hyperpermeability alteration by PDE2 inhibition is highly speculative and has to be confirmed by further functional studies.

The morphological correlate to disturbed intestinal permeability and absorption capacity in septic patients is a modified and destroyed intestinal mucosal architecture that is quantifiable by intestinal mucosal damage grading according to Chiu and colleagues [31]. By this means, we were able to demonstrate a significantly better outcome for animals treated with the ADA1/PDE2 inhibitor.

At this point we employed the well established more complex animal model of sepsis (caecal ligation after puncture) with an elevated number of individuals (n = 84) to strengthen the statement that EHNA could have beneficial effects in experimental septic conditions. Both LPS and CLP models had similar mortality rates. The data give further evidence of a survival benefit even when treatment was delayed for four hours, which is more realistic in the clinical routine and suggestive of a therapeutic potential of EHNA for treating septic conditions.

## Conclusion

In this study based on a septic animal model, we present further evidence of the beneficial effects of administering the ADA1 and PDE2 inhibitor EHNA. This effect is detectable even when EHNA is applied four hours after sepsis induction. It may therefore be a potential therapeutic option in the treatment of septic conditions – still one of medicine's big challenges.

## Key messages

EHNA treatment after experimental endotoxicosis/sepsis induction results in:

- Attenuated intestinal production of hypoxanthine (indicator of cellular energy failure);

- Decreased intestinal lactulose/L-rhamnose ratio (measure of intestinal permeability);

- Normal mucosal histology of the terminal ileum compared with an appropriate control group;

- Improved survival rate in CLP mice even when EHNA treatment was delayed for four hours.

## Abbreviations

ADA: adenosine deaminase; ANOVA: analysis of variance; APACHE: Acute Physiology and Chronic Health Evaluation; CLP: caecal ligation and puncture; EHNA: erythro-9-[2-hydroxyl-3-nonyl]-adenine; HPLC: high-performance liquid chromatography; K2HPO4: dipotassium phosphate; KH2PO4: potassium dihydrogen phosphate; LPS: lipopolysaccharide; NaCl: sodium chloride; PDE2: guanosine monophosphate-stimulated phosphodiesterase; SCID: severe combined immunodeficiency disease; SEM: standard error of the mean.

## Competing interests

The authors declare that they have no competing interests.

## Authors' contributions

NK carried out animal experiments and participated in the study design; BF participated in the design of the study, performed statistical analysis, drafted and wrote the manuscript, and prepared the figures. NK and BF contributed equal shares to this project. HW carried out animal experiments. SH and HS participated in the design of the study. HB participated in the design of the study especially the HPLC intestinal permeability experiments. CV participated in the design and co-ordination of the study. MW conceived the study idea, participated in its design and co-ordination and helped to draft the manuscript. All authors read and approved the final manuscript.
